# The correlation of asymmetrical functional connectivity with cognition and reperfusion in carotid stenosis patients

**DOI:** 10.1016/j.nicl.2018.08.011

**Published:** 2018-08-09

**Authors:** Kuo-Lun Huang, Ting-Yu Chang, Meng-Yang Ho, Wei-Hao Chen, Mei-Yu Yeh, Yeu-Jhy Chang, Ho-Fai Wong, Chien-Hung Chang, Chi-Hung Liu, Tsong-Hai Lee, Changwei W. Wu

**Affiliations:** a Department of Neurology, Linkou Chang Gung Memorial Hospital, Taoyuan, Taiwan; b College of Medicine, Chang Gung University, Taiwan; c Graduate Institute of Behavioral Sciences, Chang Gung University, Taoyuan, Taiwan; d Department of Biomedical Sciences and Engineering, National Central University, Taoyuan, Taiwan; e Graduate Institute of Mind, Brain and Consciousness, Taipei Medical University, Taipei, Taiwan; f Department of Radiology, Chang Gung Memorial Hospital, Taoyuan, Taiwan

**Keywords:** Stroke, Carotid artery stenosis, Resting-state fMRI, Functional connectivity, Hyper-connectivity

## Abstract

**Objective:**

Neural disruption and cognitive impairment have been reported in patients with carotid stenosis (CS), but carotid artery stenting (CAS) may not contribute to the cognitive recovery. Although functional hyper-connectivity is one of the physiological over-compensation phenomena in neurological diseases, the literature on the cognitive influence of functional hyper-connectivity in CS patients is limited. We aimed to investigate the longitudinal changes of hyper-connectivity after CAS and its association with cognition in CS patients.

**Methods:**

Thirteen patients with unilateral CS and 17 controls without CS were included. Cognitive function was evaluated at baseline, and resting-state functional MRI was performed 1 week before and 1 month and 1 year after CAS. Comparisons of functional connectivity (FC) between CS patients and controls in multiple brain networks were performed.

**Results:**

In patients before CAS, FC in the cerebral hemispheres ipsilateral and contralateral to CS was mainly decreased and increased, respectively, compared with normal controls. Part of the FC alterations gradually recovered to the normal condition after CAS. The stronger FC abnormality (both hypo- and hyper-connectivity compared with normal controls) was associated with poorer cognitive performances, especially in memory and executive functions.

**Conclusion:**

The study demonstrated the lateralization of hyper-connectivity and hypo-connectivity in patients with unilateral CS in contrast to the FC in normal controls. These FC alterations were associated with poor cognitive performances and tended to recover after CAS, implying that hyper-connectivity is served as a compensation for neural challenge.

## Highlights

• Cerebral hypoconnectivity on functional MRI is lateralized to carotid stenosis side.
• Cerebral hyperconnectivity is in regions contralateral to carotid stenosis side.
• Abnormal connectivity gradually returns back to normal after carotid stenting.
• Hypo- and hyper-connectivity associate with poor cognitive performances.
• Hyperconnectivity could be served as a compensation for neural challenge.

## 1 Introduction

Carotid stenosis (CS) is one of the atherosclerosis markers, and significant CS accounts for 20% to 30% of ischemic strokes (Timsit et al., 1992). Carotid revascularization with either carotid artery endarterectomy or carotid artery stenting (CAS) has shown benefits for long-term stroke prevention in patients with high-grade CS (Brott et al., 2016).

Although a series of cognitive impairments, such as attention, psychomotor speed, memory and motor dysfunction, have been attributed to cerebral hypoperfusion in patients with symptomatic and asymptomatic CS (Mathiesen et al., 2004; Silvestrini et al., 2009), cognitive changes after carotid revascularization seemed limited to a small percentage (around 10% to 15%) of the patient population with large between-subject variability (Plessers et al., 2014). The mismatches among the findings of cognitive performances after CAS could be attributed to multiple factors, such as baseline perfusion state (Chen et al., 2012), occurrence of peri-procedural vascular event (Huang et al., 2011), population type, sample size, neuroplasticity, and administered cognitive test (Plessers et al., 2014). The specific reasons leading to the poor cognitive recovery after CAS remain elusive, even though patients' physiological hypoperfusion (Chin et al., 2012) and neuronal/microstructural disconnections (Lin et al., 2016) were prominently improved.

Previous neuroimaging studies of carotid stenosis mainly reported negative cognitive inferences, such as the reduced topographical activation (Schaaf et al., 2010; Zheng et al., 2014) and the decreased inter-hemispheric connectivity associated with cognitive impairments (Cheng et al., 2012; Lin et al., 2014). Lin et al. denoted that patients with asymptomatic CS had impaired functional connectivity in the dorsal attention network (DAN), frontoparietal network (FPN) and default mode network (DMN), suggesting an association between lower connectivity and poorer cognitive performances in CS patients (Lin et al., 2014). Later on, the same group reported the elevated functional connectivity 3 months after CAS but only found associations between cognitive increments and structural connectivity after cerebrovascular revascularization (Lin et al., 2016). Conceptually, if the brain functional connectivity contributes to cognitive performances, CS patients with prolonged cognitive impairments after CAS should have sustained or worse hypo-connectivity. To date, this speculation remains unanswered and a study of longitudinal assessments of functional connectivity is warranted to verify the conjecture.

Beyond the overall connectivity reduction in CS patients, an alternative compensation phenomenon in functional connectivity has been commonly observed in a variety of neurological diseases, such as traumatic brain injury (TBI), multiple sclerosis, mild cognitive impairment (MCI) and Alzheimer's disease (AD), indicating that the hyper-connectivity could be supplementary for neurological disruptions and applicable for explaining cognitive variability among CS patients (Hillary et al., 2015). Neural compensation is the presumed underlying mechanism of hyper-connectivity for maintaining cognitive performance in patients with unilateral CS, e.g., the long-term potentiation on motor learning may be attenuated by increased gamma-aminobutyric-acid (GABA) B-mediated corticomotor excitability within the affected hemisphere (List et al., 2014). Hence, functional hyper-connectivity could plausibly coexist with neurological diseases but that is addressed less in the literature on CS (Hillary et al., 2015). Here we hypothesize that the asymmetric vascular burden in unilateral carotid stenosis not only leads to reduced ipsilateral connectivity (hypo-connectivity) but also provokes neural adaptations in the contralateral cerebral hemisphere with the occurrence of functional hyper-connectivity, which may be key to the large variability on cognitive improvements after carotid revascularization.

To test these notions, we conducted a 1-year longitudinal study to trace the progressive changes of functional connectivity in unilateral CS patients receiving CAS treatment. Multiple specific aims were tested in the current study to evaluate the connectivity alterations in unilateral CS patients following CAS: (1) the hyper-connectivity regions are mainly localized in the hemisphere contralateral to the CS side for neural compensation; (2) abnormal connectivity alterations (including both hyper- and hypo-connectivity) are negatively correlated with cognitive performance; (3) the connectivity alterations in CS patients recover after CAS.

## 2 Materials and methods

### 2.1 Participants

Twenty-two patients with significant unilateral CS (>60% on digital subtraction angiography according to the North American Symptomatic Carotid Endarterectomy Trial, NASCET, criteria) were screened for this study, and 13 of them were included based on the following exclusion criteria: 1) contralateral CS > 50%; 2) stroke attributed to the CS in the past 6 months; 3) modified Rankin Scale ≥3; 4) the presence of any expressive/receptive language disturbance; 5) co-morbidity with other neurological, degenerative, or psychiatric disorders; 6) co-morbidity with severe systemic illness including heart failure, renal insufficiency, or malignancy. In addition, 17 age- and education-matched volunteers without CS were recruited as controls. The study has been carried out in accordance with Declaration of Helsinki. Written informed consents were obtained from all subjects, and the study protocol was approved by the Institutional Review Board of Chang Gung Memorial Hospital.

### 2.2 Carotid artery stenting procedure

All patients with unilateral CS received CAS with Wallstent (Boston Scientific Corp, Natick, MA) or Precise stent (Cordis, Milpitas, CA). EPI FilterWire (Boston Scientific Corp, Natick, MA) was applied in 9 patients, and post-stenting angioplasty with balloon dilation and intra-procedural heparinization were applied in all patients. A combination of aspirin (100 mg/d) and clopidogrel (75 mg/d) was routinely administered 72 h before CAS, and was maintained for at least 3 months post-operatively before shifting to mono antiplatelet therapy. Patients were evaluated 1 day before CAS and 1 day, 1 month and 1 year after CAS by independent neurologists. One patient had very mild peri-operative ischemic stroke without an increase in the National Institute of Health Stroke Scale (NIHSS) score.

### 2.3 Cognitive performance evaluation

Neuropsychological assessments were performed in patients within 1 week before CAS in the same way as described in our previously published article (Chang et al., 2016). For evaluation of the general cognitive function, the Mini-Mental State Examination (MMSE), Raven's Standard Progressive Matrices (RSPM), and Chinese Graded Word Reading Test (CGWRT) were used. For episodic memory function evaluation, the California Verbal Learning Test-II (CVLT-II) was administered to obtain the total immediate recall score (Trial A1-A5 total). For evaluating executive functions, the Trail Making Test-A (TMT-A) and Stroop Test were used. Reaction time (RT) in the word-naming and color-naming Stroop tests was obtained, and the Stroop interference effects were defined as the RT difference between incongruent and neutral tests of the color-naming and word-naming subtests respectively. Four patients did not undergo full cognitive evaluation due to poor cooperation or illiteracy, and they were excluded from the correlation analysis between cognitive function and resting-state fMRI findings.

### 2.4 Magnetic resonance imaging (MRI) acquisition

Carotid stenosis patients underwent multiple MRI scanning sessions and the controls were scanned using the same imaging protocol once. All patients underwent brain MRI sessions 3 times: 1 week before CAS and 1 month and 1 year after CAS except for 3 patients who dropped out of 1-year imaging follow-up. The MRI protocol was carried out using a 3-Tesla scanner (Trio, Siemens, Erlangen, Germany). The anatomical images included axial T1-weighted images (spin-echo, TR/TE = 449/12 ms), axial T2-weighted images (fast spin-echo, TR/TE = 4000/90 ms), fluid-attenuated-inversion recovery (FLAIR) (TR/TE/TI = 9416/90/2200 ms), and resting-state fMRI by the single-shot gradient-echo echo planar imaging (GE-EPI) sequence (TR/TE = 2000/30 ms; flip angle = 90°; FoV = 220 mm; matrix size = 64 × 64; slice thickness = 4 mm; slice gap = 0 mm; 33 slices in total). Each GE-EPI session contained 180 time points, equivalent to 6 min in acquisition.

### 2.5 Visual rating of leukoaraiosis and cerebral infarction

The severity of cerebral infarction and leukoaraiosis was evaluated as our previous study (Huang et al., 2012). The severity of leukoaraiosis was measured over the periventricular and deep white matter areas, respectively, on the FLAIR sequence. Periventricular leukoaraiosis was graded as 0 = absence, 1=“caps or pencil-thin lining”, 2 = smooth “halo”, 3 = irregular leukoaraiosis extending into the deep white matter, and deep white matter leukoaraiosis was defined as 0 = absence, 1 = punctuate foci, 2 = beginning confluence of foci, 3 = large confluent areas. Lacunar infarcts were defined as lesions from 5 to 15 mm in diameter with the same MRI signal characteristics as cerebrospinal fluid. The severity of cerebral infarct was rated as 0 = no lesion, 1 = one focal lesion (≥5 mm), 2 = more than one focal lesion, and 3 = confluent lesions.

### 2.6 Imaging processing and analysis

All fMRI data were preprocessed using SPM8 (Wellcome Trust Centre for Neuroimaging, University College London, London, UK) and REST (Resting-State fMRI Data Analysis Toolkit) software. Both were on the platform of MATLAB version 2010 (The MathWorks, Sherborn MA, USA). The first preprocessing step was to flip the images of left-sided carotid stenosis patients, unifying the affected stenosis hemisphere located in the right hand side of the images across all patients. Other preprocessing steps included spatial realignment to the mean volume of a series of images, coregistration between anatomical and functional images, normalization to the Montreal Neurological Institute (MNI) template, and smoothness using a Gaussian filter of 6 mm FWHM. In REST, we applied detrending for removal of signal intensity drift, noise removal using nuisance regression to eliminate physiological noise of motion, white matter, and cerebrospinal fluid. The last preprocessing step was applying band-pass filtering between 0.01 and 0.08 Hz. After the preprocessing steps, we conducted seed-correlation analysis by REST. We evaluated the following 5 networks based on previous reports in CS patients: (1) DMN at the seed of the left posterior cingulate cortex (PCC, −3 -53 26); (2) sensorimotor network (SMN) at the seed of the left primary motor cortex (M1, −36 -25 57), and (3) Salience network (SAL) at the seed of the left insula (ISL, −38 20 4), (4) DAN at the seed of the left frontal eye field (FEF, −26, 6, 48), and (5) FPN at the seed of the left middle frontal gyrus (MFG, −45, 29, 32). All spherical seeds were placed to the healthy side (contralateral to the stenosis side); seed size radius was 4 mm. The functional connectivity maps were transformed into Fisher's z maps for group analysis.

### 2.7 Statistical analysis

Two-sample *t*-test and chi-square test were used to compare the demographic and cognitive function tests between patients and controls. Fisher's exact test was performed when appropriate. In the image level, the one-sample group average was based on the statistical threshold of family-wise error (FWE) corrected p < .05. Two-sample t-test was conducted for comparisons between patients and normal controls (AlphaSim corrected p < .05 with spatial autocorrelation estimations).

In the region of interest (ROI) level, two statistical methods were applied to evaluate the longitudinal changes of functional connectivity strength before and after CAS. First, we compared the functional connectivity strength of healthy controls with that of CS patients in each time point by multiple two-sample *t*-tests with Bonferroni correction. Secondly, the longitudinal changes of functional connectivity strength of CS patients were evaluated by the repeated analysis of variance (ANOVA) with Bonferroni post-hoc comparison.

Two models of partial correlation analysis were used to evaluate the association between cognitive performance and connectivity state of each ROI. Age and education effects were controlled in Model I, and the severity of leukoaraiosis and cerebral infarct by visual rating were additionally controlled in Model II. For all statistical analyses, a two-tailed p value <.05 was considered to be statistically significant.

## 3 Results

### 3.1 Demographic data

We recruited 13 patients with unilateral carotid stenosis (7 right side CS and 6 left side CS) and another 17 control subjects. There were no significant between-group differences in demographic data and general cognitive performance on MMSE, RSPM, and CGWRT tests (Table 1).

**Table 1:** Demographic characteristics of healthy controls and patients with asymptomatic unilateral carotid stenosis (CS).

	Patients, n = 13	Controls, n = 17	P value
Age, years^a^	69.3 (10.7)	62.9 (4.8)	0.07
Education, years^a^	8.2 (4.2)	10.5 (2.7)	0.08
Ipsilateral CS severity, %^a^	78.6 (11.3)	9.8 (12.8)	<0.01
Contralateral CS severity, %^a^	29.8 (11.5)	7.8 (13.8)	<0.01
Gender, male	11 (85%)	10 (59%)	0.23^b^
Hypertension	9 (69%)	8 (47%)	0.22
Diabetes mellitus	5 (38%)	4 (24%)	0.44^b^
Dyslipidemia	7 (54%)	7 (41%)	0.49
Mini-Mental State Examination^a^	24.8 (3.6)	27.4 (1.6)	0.06
Raven's Standard Progressive Matrices^a^	25.9 (9.8)	31.6 (9.9)	0.21
Chinese Graded Word Reading Test^a^	130 (26.5)	149.9 (25.4)	0.09

a Data are expressed as mean with standard deviation in parentheses.

b Analyzed by Fisher's Exact test.

### 3.2 Hypo- and hyper-connectivity between CS patients and healthy controls

**Fig. 1:**
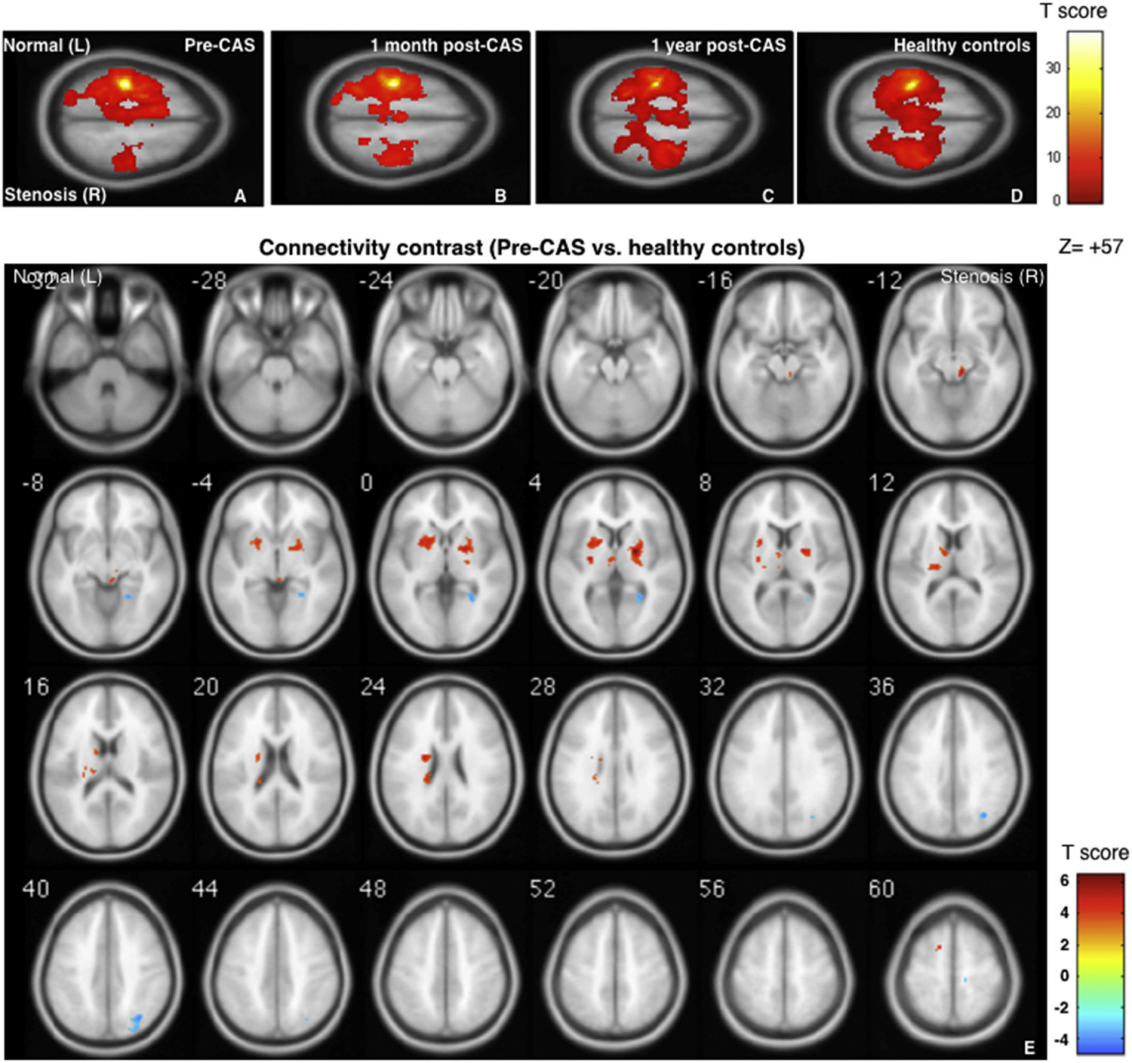
Group-level functional connectivity maps in the sensorimotor network (SMN). Four sets of one-sample SMN, seed at left primary motor cortex (M1) [−36–25 57], radius = 4, family-wise error p < .05. From left to right: carotid stenosis patients before carotid artery stenting (CAS) (A), 1 month after CAS (B), 1 year after CAS (C), and healthy controls (HC) (D). Two-sample contrast map (Pre-CAS > HC) with AlphaSim corrected p < .05. Red blobs show hyper-connectivity and blue blobs show hypo-connectivity (E). (For interpretation of the references to color in this figure legend, the reader is referred to the web version of this article.)

**Fig. 2:**
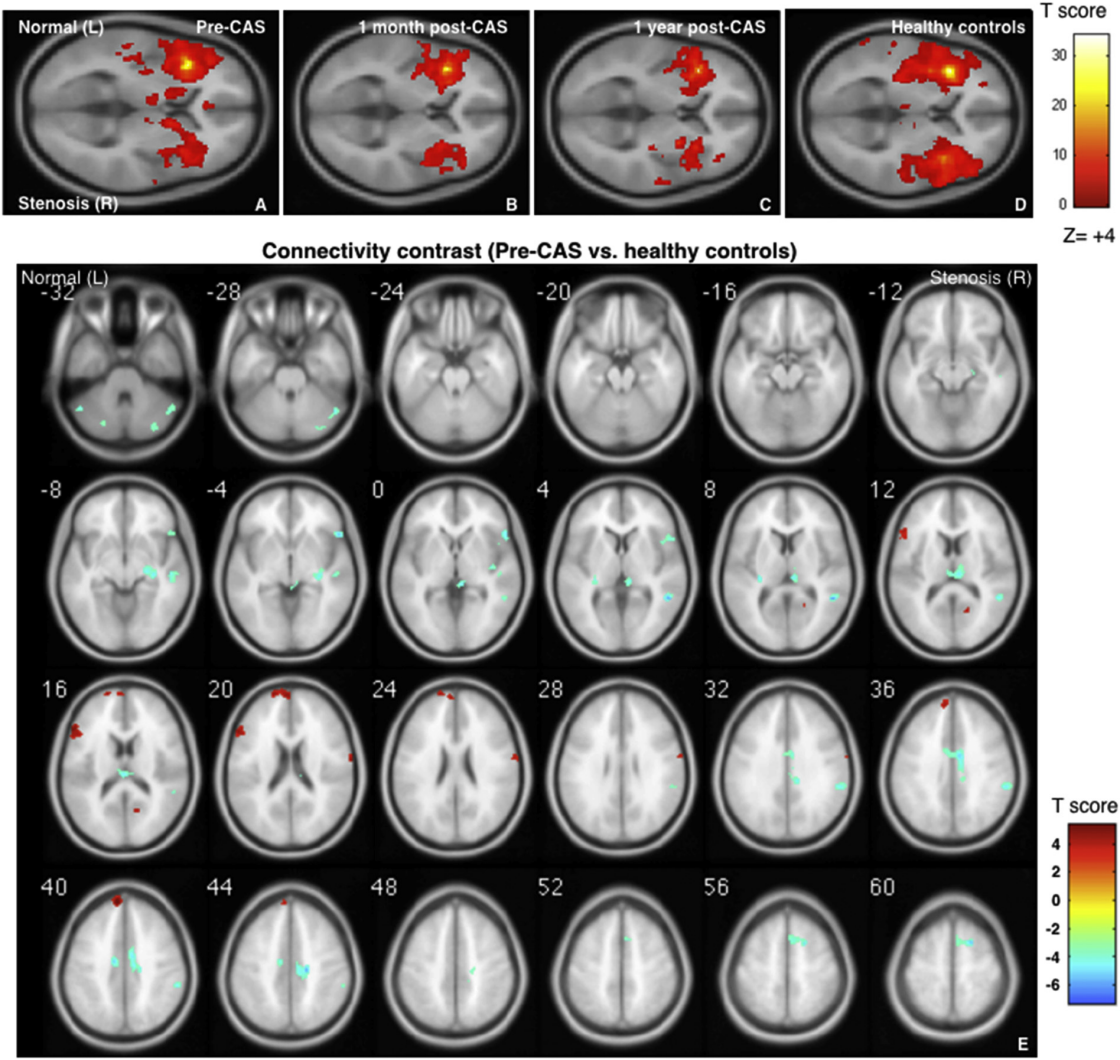
Group-level functional connectivity maps in the salience network (SAL). Four sets of one-sample SAL, seed at left insula (ISL) [−38 20 4], radius = 4, family-wise error p < .05. From left to right: carotid stenosis patients before carotid artery stenting (CAS) (A), 1 month after CAS (B), 1 year after CAS (C), and healthy controls (HC) (D). Two-sample contrast map (Pre-CAS > HC) with AlphaSim corrected p < .05. Red blobs show hyper-connectivity and blue blobs show hypo-connectivity (E). (For interpretation of the references to color in this figure legend, the reader is referred to the web version of this article.)

In the patient group before CAS, the stenosis ipsilateral side (right; with seeding at the stenosis contralateral side, left) showed asymmetrical hypo-connectivity among the 5 selected networks. Here the hypo- and hyper-connectivity of one specific network in CS patients was defined as the abnormal connectivity strengths in comparison with those in healthy controls. One month and 1 year after CAS, the inter-hemispheric functional connectivity gradually became symmetrical, toward the presentations in the healthy controls as shown in Fig. 1A to 1D for the SMN and Fig. 2A to 2D for the SAL (FWE-corrected p < .05). When comparing SMN connectivity between healthy controls and CS patients before CAS (Fig. 1E, AlphaSim-corrected p < .05), negative connectivity contrasts (blue blobs) indicated hypo-connectivity before CAS, which was observed at the premotor cortex, superior occipital gyrus and precuneus on the stenosis ipsilateral side (R). In the SAL contrast map (Fig. 2E), hypo-connectivity spots were observed at the right premotor, inferior orbital frontal, supramarginal, middle temporal and cingular gyri.

For hyper-connectivity contrasts, Fig. 1E also indicates the hyper-connectivity regions in the SMN contrast maps before CAS (red blobs), localized at the putamen, caudate, thalamus, and premotor cortex in the healthy side and the pallidum in the ipsilateral side. The hyper-connectivity regions in the SAL network were observed at the prefrontal and inferior frontal cortices in the healthy side; and the calcarine and primary motor cortices in the stenosis side (Fig. 2E). In general, the laterality of hypo-connectivity was prone to the stenosis side, whereas the hyper-connectivity phenomenon was mostly noted in the SMN and SAL connectivity before CAS, being frequently observed in the contralateral hemisphere (the healthy side). It was noted that no prominent connectivity changes were noted in the DMN (Supplemental Fig. S1), and only the stenosis-side hypo-connectivity was found in the angular gyrus of the DAN and the fusiform of the FPN (Supplemental Figs. S2 and S3, respectively). The coordinates of these spots are summarized in Supplemental Table S1.

### 3.3 Association between cognitive performance and connectivity alteration

**Fig. 3:**
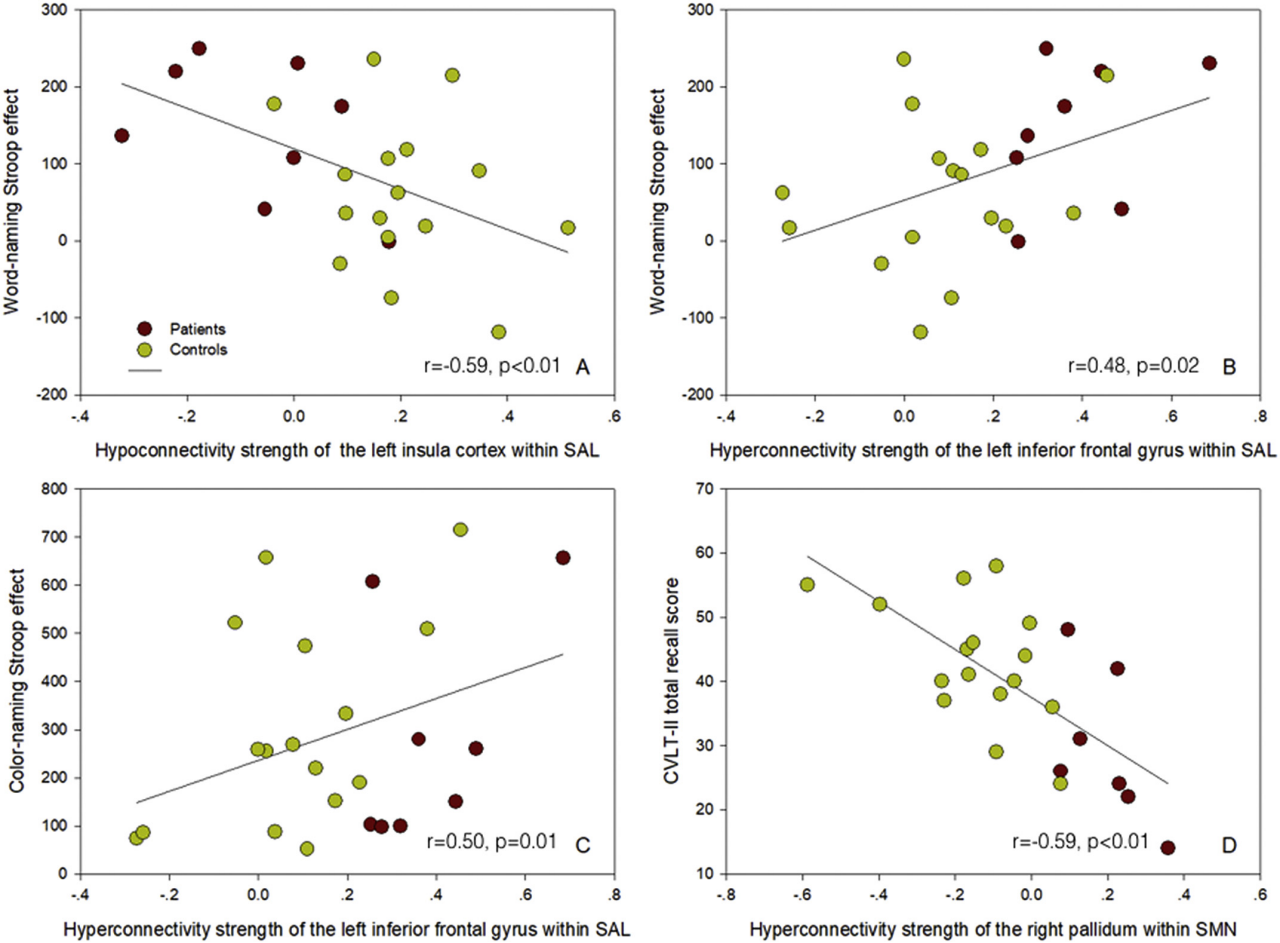
The correlation of the connectivity strength in the sensorimotor network (SMN) and salience network (SAL) with cognitive performance. The correlation of the hypo-connectivity strength of the left insula gyrus in the SAL with the word-naming Stroop effect (A). The correlations of the hyper-connectivity strength of the left inferior frontal gyrus in the SAL with the word-naming (B) and color-naming Stroop effects (C). The correlation of the hyper-connectivity strength of the right pallidum in the SMN with the California Verbal Learning Test-II (CVLT-II) total recall score (D).

The correlation between cognitive performance and the connectivity strength of each ROI in the SMN and SAL networks before stenting is summarized in Table 2. Generally, low connectivity strength in hypo-connectivity regions was associated with worse cognitive performance, as depicted in Fig. 3A for the association between the word-naming Stroop effect and the left insula gyrus in the SAL network. As a compensatory mechanism, high connectivity strength in hyper-connectivity ROIs was associated with worse cognitive performance, for example 1) the associations of the word-naming (Fig. 3B) and color-naming Stroop effects (Fig. 3C) with the inferior frontal gyrus-insula SAL hyper-connectivity strength in the healthy side and 2) the association between the CVLT-II total recall score and the M1-pallidum SMN connectivity in the stenosis side (Fig. 3D). Furthermore, lower education attainment and older age was associated with more positive connectivity strength in hyper-connectivity, and with more negative connectivity strength in hypo-connectivity (Supplemental Table S2).

**Table 2:** The correlation between cognitive performance and connectivity strength of each region of interest (ROI) in the sensorimotor network (SMN) and salience network (SAL) before carotid artery stenting.

	MMSE	MoCA	CVLT_Total	TMT-A	Stroop_W Inc	Stroop_W Effect	Stroop_C Inc	Stroop_C Effect
Hyper-connectivity ROI in SMN								
Pallidum, R	−0.32	−0.25	−0.59⁎, †	0.28	0.33	0.18	0.43⁎	−0.03
Putamen, L	0.02	−0.07	−0.35	0.04	0.06	0.01	0.43⁎	0.14
Caudate, L	0.14	−0.19	−0.29	0.19	0.22	0.08	0.48⁎, †	0.26
Premotor, L	−0.13	0.06	−0.45⁎	0.03	0.28	0.34	0.1	−0.18
Thalamus, L	0.05	−0.13	−0.11	0.1	−0.03	−0.07	0.62⁎, †	0.41†

Hypo-connectivity ROI in SMN								
Premotor, R	0.24	0.13	0.02	−0.1	−0.08	−0.09	−0.46⁎	−0.32
Occipital_Sup, R	0.14	0.08	0.39	−0.18	−0.34	−0.66⁎, †	−0.2	0.06
Precuneus, R	0.08	−0.01	0.25	−0.09	−0.27	−0.37	−0.54⁎, †	−0.29

Hyper-connectivity ROI in SAL								
Prefrontal, BA 9, L	−0.25	−0.08	−0.22	0.15	0.47⁎, †	0.54⁎, †	0.16	0.02
Prefrontal, BA 10, L	−0.27	0.16	−0.14	0.15	0.45⁎, †	0.54⁎, †	0.34	0.15
Inf. frontal, BA 45, L	0.16	−0.27†	−0.37	0	0.15	0.48⁎, †	0.53⁎, †	0.50⁎, †
Postcentral, R	0.11	−0.42	−0.19	0.15	0.15	0.28	0.41	0.28
Calcarine, R	−0.36	−0.26	0.06	0.16	0.22	0.21	0.28	0.13

Hypo-connectivity ROI in SAL								
Insula, L	0.46⁎	0.2	0.29	−0.53⁎, †	−0.58⁎, †	−0.59⁎, †	−0.44⁎	−0.03
Cingulum_Mid, R	0.24	0.50⁎, †	0.32	−0.32	−0.34	−0.2	0.08	0.33
Temporal_Mid, R	−0.07	0.21	0.14	−0.04	−0.08	−0.02	−0.16	0
Premotor, R	0.17	0.05	0.36	−0.18	−0.19	−0.26	−0.21	0.2
Inf. Orb. frontal, BA 47, R	0.24	0.1	0.03	−0.09	−0.06	−0.12	−0.06	0.15
Hippocampus, R	0.16	−0.25	0.18	−0.08	−0.25	−0.59⁎, †	−0.45⁎	−0.2
SupraMarginal, R	0.16	0.2	0.25	−0.27	−0.21	−0.19	0.01	0.33

MMSE indicates Mini-Mental State Examination; MoCA, Montreal Cognitive Assessment; TMT-A, Trail-Making Test A; CVLT_Total, California Verbal Learning Test total recall score; Stroop_W, word-naming Stroop test; Stroop_C, color-naming Stroop test; Con, congruent; Neu, neutral; Inc., incongruent; Inf, inferior; Orb, orbital.

⁎ p < .05 after adjusted for age and education.

† p < .05 after adjusted for age, education, leukoaraiosis, and infarct severity on MRI.

### 3.4 Recovery of connectivity alterations after CAS

The longitudinal assessments of hyper-connectivity or hypo-connectivity within the CS group are listed in Table 3, and the p values for multiple two-sample *t*-test comparisons of connectivity strength between controls and CS patients were separately listed in Supplemental Table S3 for brevity. The corresponding line graphs for individual data were shown in Supplemental Figs. S4 to S7. In the repeated analysis of variance for CS patients, there was moderate to significant trend for post-CAS connectivity attenuation in some regions of SMN (right pallidum, p = .06; right occipital_sup, p = .022) and SAL (left insula, p = .075, right temporal_mid, p = .003; right inferior orbital frontal cortex, p = .092; right hippocampus, p = .003). In general, three scenarios were discovered. First, the connectivity strengths in patients before CAS were significantly different from the healthy controls, and the difference in part of the connections attenuated 1 month after CAS (e.g., contralateral premotor-contralateral M1 hyper-connectivity). Second, several other connections remained sustained at the abnormal level 1 month after CAS, but they gradually recovered back to the normal state after 1 year (e.g., contralateral caudate-contralateral M1 hyper-connectivity and ipsilateral hippocampus-contralateral insula hypo-connectivity). Third, part of the connections did not return back to normal state even in the 1-year assessment (e.g., contralateral thalamus-contralateral M1 hyper-connectivity). In summary, the abnormal connectivity did not fully recover back to the normal status immediately after CAS treatment.

**Table 3:** The correlation coefficients of extracted time courses in the ROIs of the SMN and SAL among the healthy controls and patients 1 week before, and 1 month and 1 year after CAS.

	Before CAS	1 month after CAS	1 year after CAS	Healthy controls
Hyper-connectivity ROIs in SMN				
Pallidum, R	0.15 (0.12)⁎	0.08 (0.13)⁎	0.01 (0.16)	−0.14 (0.16)
Putamen, L	0.15 (0.11)⁎	0.06 (0.16)⁎	0.04 (0.20)	−0.09 (0.14)
Caudate, L	0.17 (0.15)⁎	0.12 (0.17)⁎	0.06 (0.31)	−0.12 (0.20)
Premotor, L	0.30 (0.14)⁎	0.21 (0.26)	0.19 (0.27)	0.04 (0.16)
Thalamus, L	0.15 (0.14)⁎	0.13 (0.17)⁎	0.08 (0.15)⁎	−0.09 (0.16)

Hypo-connectivity ROIs in SMN				
Premotor, R	0.09 (0.21)⁎	0.18 (0.25)⁎	0.31 (0.38)	0.45 (0.26)
Occipital_Sup, R	−0.09 (0.16)⁎	0.06 (0.14)†	0.06 (0.16)	0.14 (0.14)
Precuneus, R	−0.12 (0.14)⁎	−0.12 (0.30)⁎	−0.05 (0.32)	0.22 (0.28)

Hyper-connectivity ROIs in SAL				
Prefrontal, BA 9, L	0.12 (0.18)⁎	0.00 (0.24)	0.01 (0.28)	−0.18 (0.17)
Prefrontal, BA 10, L	0.16 (0.19)⁎	−0.01 (0.28)	0.04 (0.25)	−0.12 (0.15)
Inf. frontal, BA 45, L	0.38 (0.17)⁎	0.22 (0.23)	0.25 (0.25)	0.07 (0.19)
Postcentral, R	0.17 (0.17)⁎	0.06 (0.14)⁎	0.08 (0.25)	−0.07 (0.12)
Calcarine, R	0.07 (0.19)⁎	0.07 (0.31)⁎	−0.05 (0.23)	−0.20 (0.17)

Hypo-connectivity ROIs in SAL				
Insula, L	−0.08 (0.17)⁎	0.11 (0.26)	0.07 (0.17)	0.19 (0.14)
Cingulum_Mid, R	−0.12 (0.12)⁎	−0.05 (0.20)⁎	−0.07 (0.13)⁎	0.18 (0.12)
Temporal_Mid, R	−0.14 (0.10)⁎	−0.01 (0.13)⁎, †	0.04 (0.12)†	0.12 (0.12)
Premotor, R	−0.05 (0.14)⁎	0.02 (0.21)⁎	0.07 (0.19)⁎	0.26 (0.17)
Inf. Orb. frontal, BA 47, R	0.11 (0.13)⁎	0.27 (0.24)	0.21 (0.16)⁎	0.42 (0.18)
Hippocampus, R	−0.12 (0.16)⁎	−0.02 (0.15)⁎	0.14 (0.18)†	0.17 (0.09)
Supra Marginal, R	0.04 (0.18)⁎	0.11 (0.26)⁎	0.04 (0.19)⁎	0.35 (0.18)

Data are expressed as mean with standard deviation in parentheses.

ROIs indicates regions of interest; SMN, sensorimotor network; SAL, salience network; CAS, carotid artery stenting; Inf, inferior; Orb, orbital.

⁎ Significant difference versus healthy controls by multiple t-test with Bonferroni correction.

† Post hoc comparison with Bonferroni correction shows significant difference versus the Before CAS condition in the repeated analysis of variance (ANOVA).

## 4 Discussion

Carotid stenosis is not only associated with ischemic stroke risk, but also linked to cognitive impairments. The asymmetrical neural and microstructural disconnection in patients with unilateral CS have been shown to be related to cognitive performances (Cheng et al., 2012; Lin et al., 2014), and similar findings were also noted in our study that most of the regional hypo-connectivity was located in the cerebral hemisphere ipsilateral to the CS side and worse hypo-connectivity strength was associated with poorer cognitive performances. On the other hand, SMN and SAL hyper-connectivity was found in the contralateral cerebral hemisphere, and may serve as a compensatory reaction to the hostile cognitive influence from CS, in which the worse cognitive performance came with stronger hyper-connectivity. Furthermore, these abnormal connectivity alterations tended to gradually attenuate after CAS, although part of the abnormal connections could sustain for >1 year. In summary, our study demonstrated the compensatory neural adaptations of CS patients in relation to cognition, and also evaluated their longitudinal post-CAS changes of functional connectivity.

### 4.1 Hyper-connectivity

Hyper-connectivity is a common reaction to neural disruption, and its occurrence and strength are associated with the interactions between situational demands, the degree of challenge posed by neurological disruption and resources availability (Hillary et al., 2015). The hyper-connectivity patterns would vary according to the lesion locations in different neurological diseases, and the increased connectivity in additional brain regions could be a result of cortical dedifferentiation to maintain brain function (Steffener and Stern, 2012). In Hillary's review, increased connectivity was observed in patients with TBI and multiple sclerosis, while there was a shift to diminished connectivity as degeneration progresses in patients with MCI and Alzheimer's disease (Hillary et al., 2015). Unilateral CS is a unique scenario of asymmetrical neural disruption, and our study findings suggest that there would be laterality of hyper-connectivity and hypo-connectivity when most of the neural challenge is placed in one cerebral hemisphere. Similar asymmetrical hyper-connectivity patterns were also noted in a TBI rat study carried out with the unilateral controlled cortical impact injury model, in which the increased connectivity after TBI was largely in the subcortical and contral-lesional cortex (Harris et al., 2016).

Patients with increased hyper-connectivity often showed inefficient cognitive processing ability, instead of reflecting the inability of cognitive processes (Hillary et al., 2015). In patients with moderate to severe TBI, increased connectivity during rest between task-related ROIs has been associated with worse performance on a measure of the set-shifting task (Bernier, 2017). Similar findings were also noted in our study as poor executive function was associated with hyper-connectivity in the SMN and SAL networks and poor semantic memory performance was associated with hyper-connectivity in the SMN network. These hyper-connectivity phenomena did not show a direct link with executive control and the hippocampus network, implying that hyper-connectivity plays a role in mediating cross-network functionality. Therefore, such cross-network resource mediation under hypoperfusion could be taken as one compensation factor for maintaining cognitive performance (Steffener and Stern, 2012).

### 4.2 Connectivity alterations over time

Most of the hyper-connectivity observations are based on post-injury models, because the pre-injury connectivity state is usually unavailable. In contrast, patients with CS can undergo elective carotid revascularization, making assessment of the connectivity states before and after revascularization possible. In our study, there was a tendency for both the hyper-connectivity and hypo-connectivity strength to continuously attenuate toward the connectivity state of the heathy controls 1 month and 1 year after CAS. In fact, such reciprocal regression in hyper-connectivity and hypo-connectivity would further underpin the notion that hyper-connectivity is an auto-regulative reaction to neural disruption and its activity is associated with the degree of neural challenge. Furthermore, the hemodynamic disturbance can be restored soon after CAS (Chin et al., 2012), and connectivity changes 1 year after CAS could be attributed to neuroplasticity. However, cerebral perfusion was not evaluated in our subject pool, and the influence of hypoperfusion correction on the degrees of connectivity attenuation after CAS remained to be determined. With regard to the tendency of hyper-connectivity and hypo-connectivity post-CAS attenuation toward the connectivity state of healthy subjects, the possibility of “regression to the mean” should be considered as our study did not compare the longitudinal effects between CS patients with and without receiving CAS treatment. Furthermore, lifestyle modification has been shown associated with function connectivity changes (Topiwala et al., 2018). However, lifestyle changes after CAS were not formally recorded in our patients, and this confounding factor should be taken into account regarding to the post-CAS functional connectivity changes.

The hyper-connectivity ROIs in the SMN network were mainly located in the subcortical nuclei, including the caudate, pallidum, putamen and thalamus. Interestingly, these hyper-connectivity loci are part of the frontal-subcortical circuit, which is involved in aspects of planning, working memory, rule-based learning and the decision threshold in reaction time tasks (Bonelli and Cummings, 2007). These previous results are compatible with our findings that the connectivity strength in the SMN hyper-connectivity spots were correlated with the CVLT memory test and color-naming Stroop test results. As for the connectivity strength of the SAL hyper-connectivity and hypo-connectivity ROIs, they were mostly correlated with the Stroop test results, not the CVLT memory test results. Such findings are consistent with the view that the SAL network responds to behaviorally salient stimuli and helps determine which inputs are more likely to capture attention (Ham et al., 2013; Uddin, 2015).

### 4.3 Confounding factors

In our study, education attainment was correlated with the strength of hyper-connectivity and hypo-connectivity. Subjects with lower education attainment had higher hyper-connectivity and worse hypo-connectivity, reflecting that they may have lower neural resources and that the presence of hyper-connectivity and hypo-connectivity are reciprocal dynamic changes. Education attainment is often deemed as the proxy measure for cognitive reserve, and subjects with higher cognitive reserve could tolerate more brain pathology and bypass the needs to recruit compensatory resources (Steffener and Stern, 2012; Watson and Joyce, 2015). Furthermore, the correlations between education attainment and connectivity alterations may suggest that the observed hyper-connectivity and hypo-connectivity in CS patients is related to neuronal activity and not simply the blood flow effect.

As for the influence of unilateral CS on functional connectivity, decreased intra- and inter-hemispheric connectivity was noted in the ipsilateral cerebral hemisphere (Cheng et al., 2012). On the other hand, Lin et al. once observed the ipsilateral superior temporal lobule hyper-connectivity of the frontoparietal network in CS patients, and attributed it to a less anti-correlated activity rather than a compensatory activity (Lin et al., 2014). Similar findings on hypo-connectivity were noted in our study, and, furthermore, there was more hyper-connectivity in the contralateral cerebral hemisphere. The discrepancy in the hyper-connectivity findings might be attributed to differences in the sample and seed selections. Our patients had lower education attainment and lower MMSE score, and their low cognitive reserve might have high drive to recruit additional brain regions to cope with the asymmetrical vascular burden. Furthermore, the seeds for extracting time series correlation coefficients were placed in the contralateral cerebral hemisphere in our study because the connectivity of the spared cerebral hemisphere was the main focus in our analysis, while the seeds were placed in the ipsilateral cerebral hemisphere in previous studies (Lin et al., 2016; Cheng et al., 2012; Lin et al., 2014).

There were limitations in our study. First, structural connectivity was not examined. Diminished mean fractional anisotropy was reported to be associated with functional hypo-connectivity (Chin et al., 2012). How the functional hyper-connectivity interplays with structural changes requires further investigation. Secondly, there were correlations of cognitive performance with hyper-connectivity and hypo-connectivity strength. However, the sample size was relatively small, and a larger sample size is necessary to replicate the study results. Thirdly, there was temporal attenuation in functional hyper-connectivity and hypo-connectivity after CAS, but we were unable to estimate the influence of connectivity changes on cognitive performance after CAS because the cognitive test results in the follow-up visits were not accessed. It requires longitudinal studies to determine whether such connectivity attenuations are associated with long-term changes in cognitive performance.

## 5 Summary

In conclusion, we found patients with unilateral CS have lateralized functional connectivity in the SMN and SAL networks. The prolonged and lateralized hyper-connectivity may act as the compensation factor in neuroplasticity, affecting the cognitive performance in patients with unilateral CS. Our finding is the first study to investigate the compensatory neural adaptations to the influence of CS before and after CAS.

## Funding

This study was carried out under the grants from the Ministry of Science and Technology, Taiwan (Grants MOST 106-2314-B-182A-074105-2628-B-038-013-MY3), and the Research Fund of Chang Gung Memorial Hospital (CMRPG3B0331-3, CMRPG3F2182, BMRPD69).

## Declaration of interest

None.

## Appendix A Supplementary data

Supplementary material Image 1
