# Supplementary material for: The correlation of asymmetrical functional connectivity with cognition and reperfusion in carotid stenosis patients
Source: Neuroimage Clin. 2018 Aug 9;20:476–84. doi: 10.1016/j.nicl.2018.08.011 (PMC6098231; doi:10.1016/j.nicl.2018.08.011)
Supplement: Supplementary file 1 — Supplementary material [file mmc1.docx]

**Supplemental Figures**

**Figure S1.** Group-level functional connectivity maps in the default mode network (DMN). Four sets of one-sample DMN, seed at left posterior cingulate cortex [-3 -53 26], radius=4, uncorrected p<0.001, threshold = 40 voxels. From left to right: carotid stenosis patients before carotid artery stenting (CAS) (A), 1 month after CAS (B), 1 year after CAS (C), and healthy controls (HC) (D). Two-sample contrast map (Pre-CAS > HC) shows no significant hypo- or hyper-connectivity spot (E).


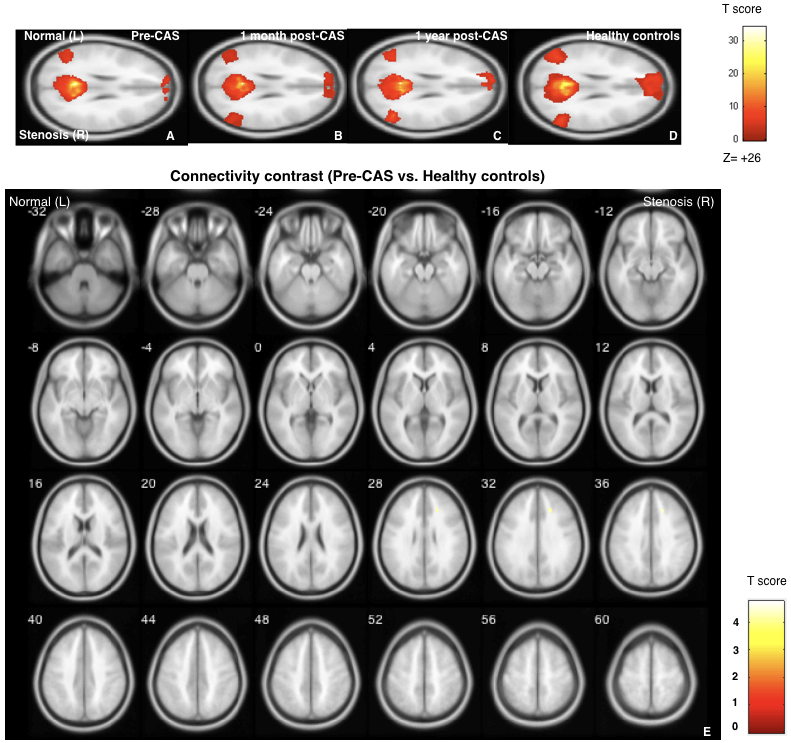


**Figure S2.** Group-level functional connectivity maps in the dorsal attention network (DAN). Four sets of one-sample DAN, seed at left frontal [-26 6 48], radius=4, uncorrected p<0.001, threshold = 40 voxels. From left to right: carotid stenosis patients before carotid artery stenting (CAS) (A), 1 month after CAS (B), 1 year after CAS (C), and healthy controls (HC) (D). Two-sample contrast map (Pre-CAS > HC) with Zmap cluster=53 voxels. Yellow blobs show hypo-connectivity (E).


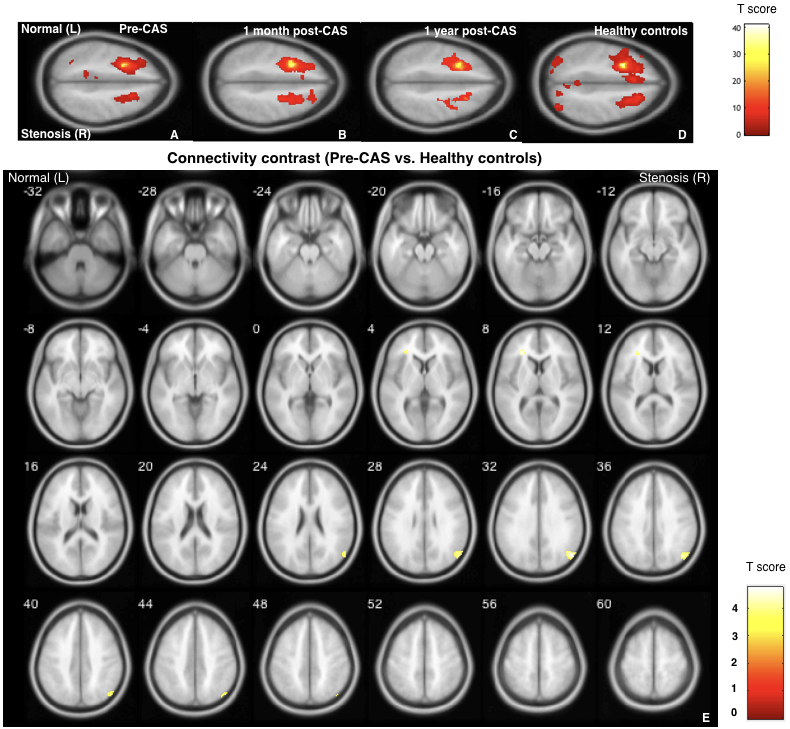


**Figure S3.** Group-level functional connectivity maps in the frontoparietal network (FPN). Four sets of one-sample FPN, seed at left middle frontal gyrus [-45 29 32], radius=4, uncorrected p<0.001, threshold = 40 voxels. From left to right: carotid stenosis patients before carotid artery stenting (CAS) (A), 1 month after CAS (B), 1 year after CAS (C), and healthy controls (HC) (D). Two-sample contrast map (Pre-CAS > HC) with Zmap cluster=56 voxels. Yellow blobs show hypo-connectivity (E).


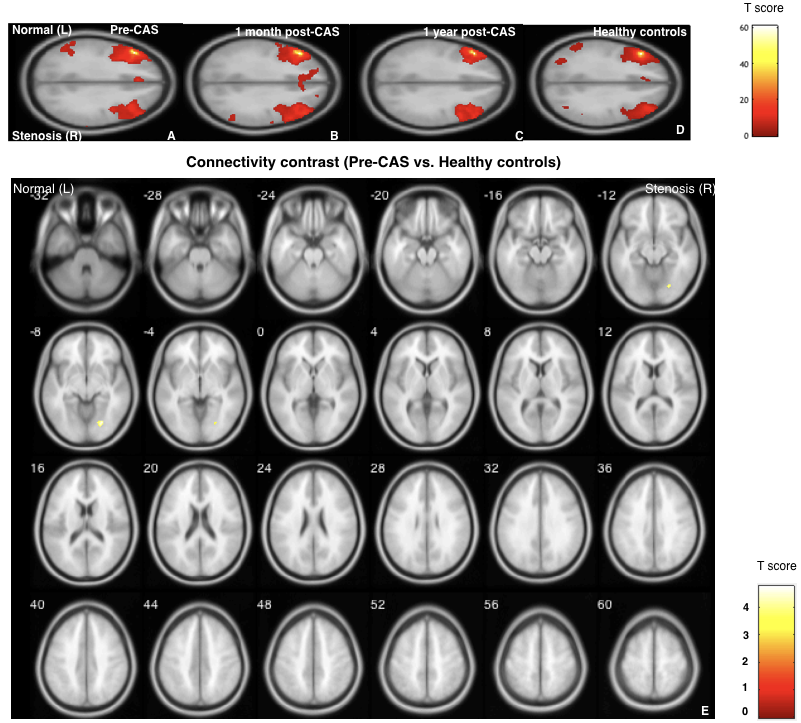


**Figure S4. The correlation coefficients (connectivity strength) in the hyper-connectivity ROIs in the sensorimotor network for each individual.**

**
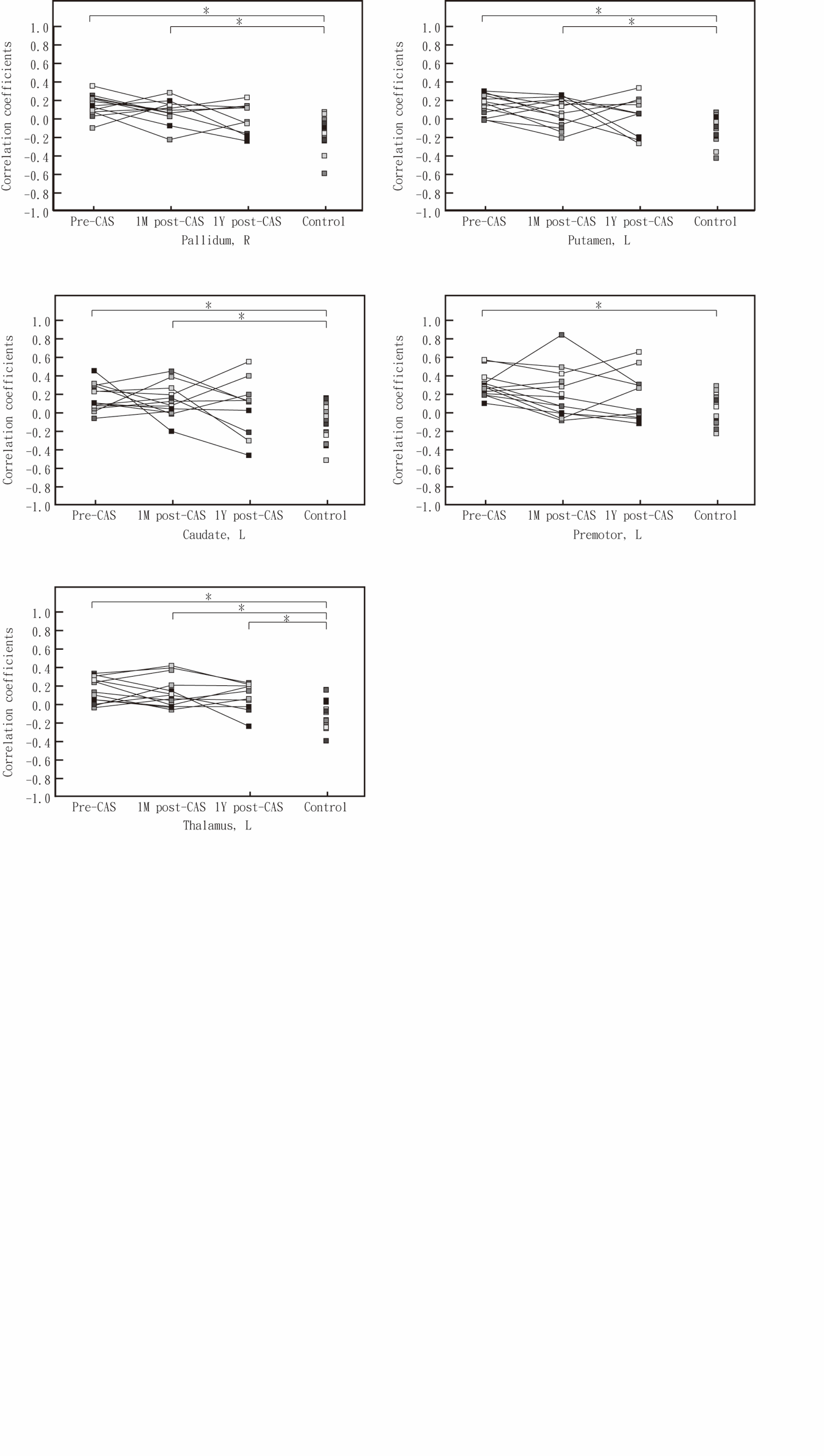
**

*Significant difference versus healthy controls by multiple t-test with Bonferroni correction.

**Figure S5. The correlation coefficients (connectivity strength) in the hypo-connectivity ROIs in the sensorimotor network for each individual.**

**
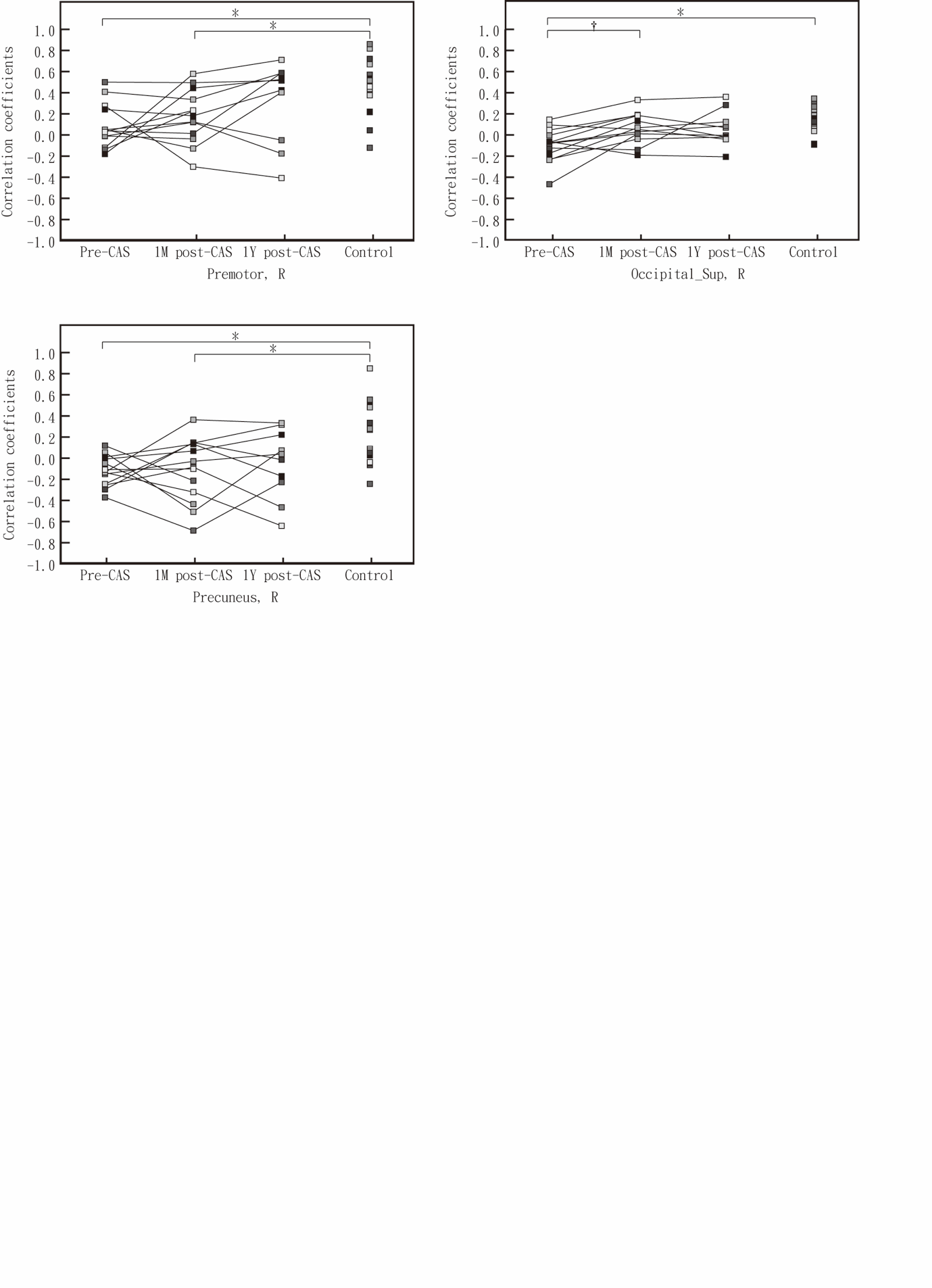
**

*Significant difference versus healthy controls by multiple t-test with Bonferroni correction. †Post hoc comparison with Bonferroni correction shows significant difference versus the Before CAS condition in the repeated analysis of variance (ANOVA).

**Figure S6. The correlation coefficients (connectivity strength) in the hyper-connectivity ROIs in the salience network for each individual.**

**
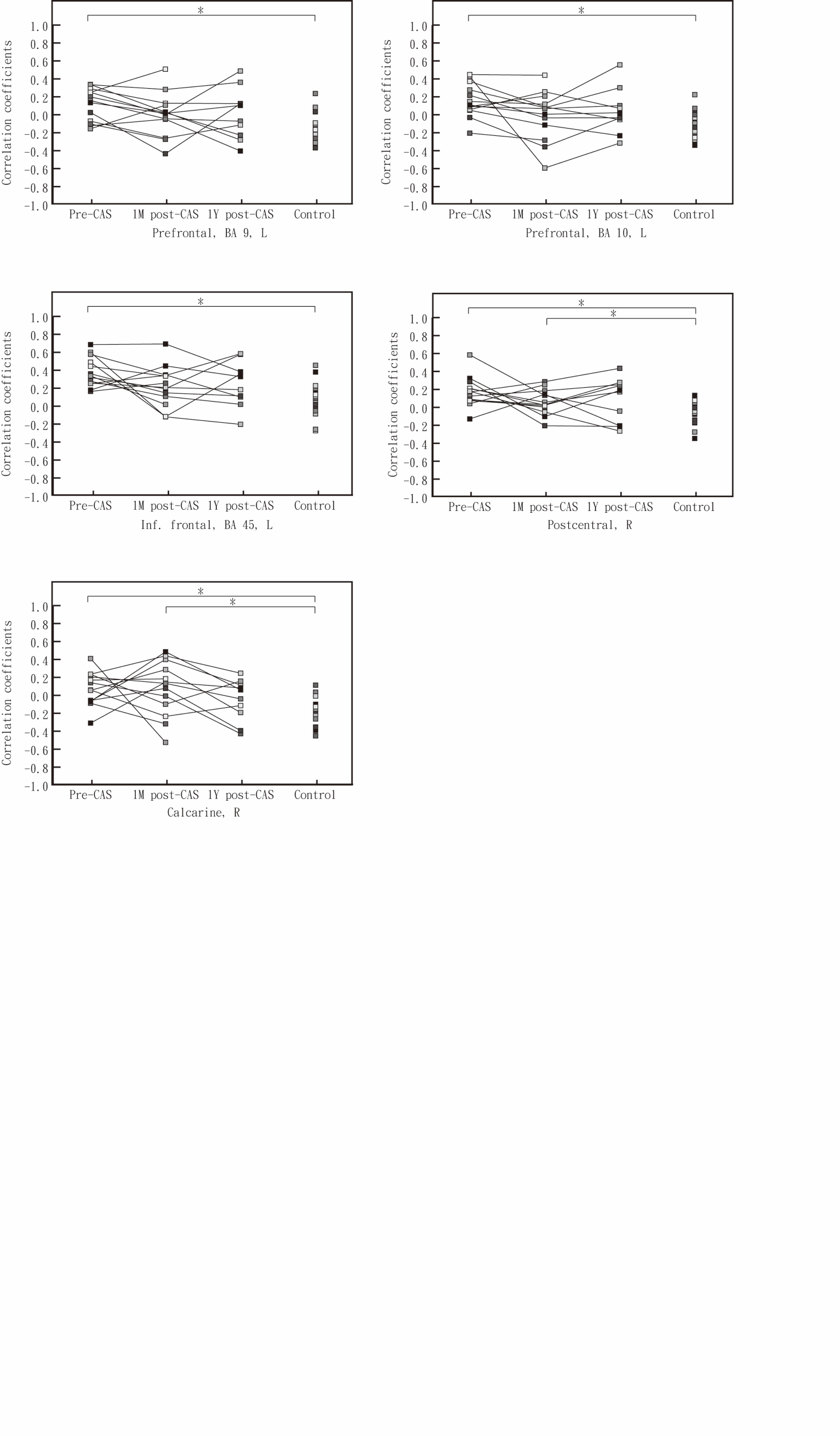
**

*Significant difference versus healthy controls by multiple t-test with Bonferroni correction.

**Figure S7. The correlation coefficients (connectivity strength) of the hypo-connectivity ROIs in the salience network for each individual.**

**
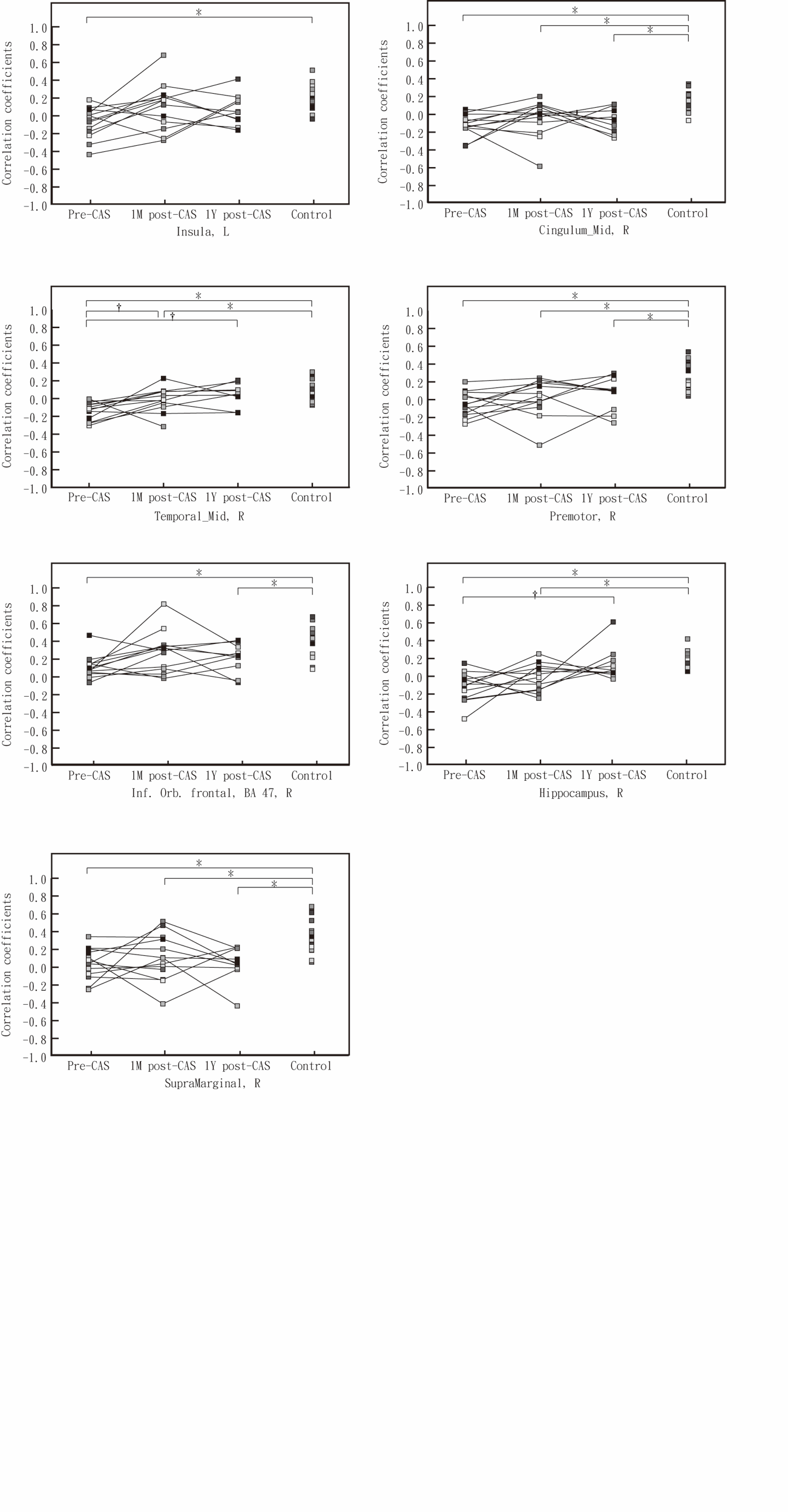
**

*Significant difference versus healthy controls by multiple t-test with Bonferroni correction. †Post hoc comparison with Bonferroni correction shows significant difference versus the Before CAS condition in the repeated analysis of variance (ANOVA).

**Supplemental Tables**

| **Table S1. List of regions of interest (ROIs) for extracting time courses in the sensorimotor network (SMN), salience network (SAL), dorsal attention network (DAN), and frontoparietal network (FPN)** | | | | | |
| --- | --- | --- | --- | --- | --- |
| Regions of interest | Coordinates (MNI) | | | Cluster size | T score |
|  | x | y | z |  |  |
| Hyper-connectivity ROIs in SMN (Pre-stent > HC) | | | | | |
| Pallidum (R) | 22 | -4 | 4 | 320 | 6.48 |
| Putamen (L) | -28 | 8 | 2 | 221 | 4.99 |
| Caudate (L) | -10 | -2 | 12 | 74 | 4.22 |
| Premotor (L) | -14 | 4 | 64 | 45 | 5.13 |
| Thalamus (L) | -18 | -20 | 12 | 63 | 4.34 |
| Hypo-connectivity ROIs in SMN (Pre-stent < HC) | | | | | |
| Premotor (R) | 12 | -24 | 64 | 49 | 4.84 |
| Superior occipital gyrus (R) | 34 | -68 | 40 | 94 | 4.36 |
| Precuneus (R) | 26 | -52 | 2 | 82 | 4.12 |
| Hyper-connectivity ROIs in SAL (Pre-stent > HC) | | | | | |
| Prefrontal, BA 9 (L) | -8 | 54 | 40 | 85 | 5.38 |
| Prefrontal, BA 10 (L) | -4 | 62 | 22 | 125 | 4.66 |
| Inf. Frontal, BA 45 (L) | -56 | 24 | 18 | 154 | 4.94 |
| Postcentral (R) | 68 | -10 | 20 | 62 | 4.84 |
| Calcarine (R) | 14 | -62 | 14 | 32 | 4.06 |
| Hypo-connectivity ROIs in SAL (Pre-stent < HC) | | | | | |
| Insula (L) | -30 | -26 | 8 | 34 | 4.72 |
| Cingulum_Mid (R) | 18 | -20 | 44 | 434 | 6.05 |
| Temporal_Mid (R) | 52 | -48 | 6 | 139 | 7.24 |
| Premotor (R) | 24 | 6 | 70 | 344 | 6.15 |
| Inf. Orb. Frontal, BA 47 (R) | 52 | 22 | -4 | 121 | 5.46 |
| Hippocampus (R) | 32 | -22 | -6 | 149 | 5.06 |
| SupraMarginal (R) | 58 | -38 | 34 | 119 | 4.88 |
| Hypo-connectivity ROIs in DAN (Pre-stent < HC) | | | | | |
| Angular (R) | 50 | -68 | 32 | 248 | 4.69 |
| Hypo-connectivity ROIs in FPN (Pre-stent < HC) | | | | | |
| Fusiform (R) | 24 | -74 | -10 | 58 | 4.63 |
| AlphaSim-Corrected p<0.01 [uncorrected p<0.001 and cluster size SMN(41) SAL(24) ]. | | | | | |

HC indicates healthy control.

| **Table S2. The correlations of education and age with the connectivity strength of each region of interest (ROI) in the sensorimotor network (SMN) and salience network (SAL)** | | | | | |
| --- | --- | --- | --- | --- | --- |
| Regions of interest | Education | |  | Age | |
|  | Correlation coefficient | p value |  | Correlation coefficient | p value |
| Hyper-connectivity ROIs in SMN | | | | | |
| Pallidum_R | -0.28467 | 0.1273 |  | 0.18548 | 0.3264 |
| Putamen_L | -0.31546 | 0.0895 |  | 0.1121 | 0.5554 |
| Caudate_L | -0.40397 | 0.0268 |  | 0.22205 | 0.2383 |
| Premotor_L | -0.41185 | 0.0237 |  | 0.36594 | 0.0467 |
| Thalamus_L | -0.24855 | 0.1854 |  | 0.23723 | 0.2069 |
| Hypo-connectivity ROIs in SMN | | | | | |
| Premotor_R | 0.09668 | 0.6113 |  | -0.0498 | 0.7938 |
| Occipital_Sup_R | 0.33371 | 0.0715 |  | -0.21044 | 0.2643 |
| Precuneus_R | 0.19376 | 0.3049 |  | -0.22579 | 0.2302 |
| Hyper-connectivity ROIs in SAL | | | | | |
| Prefontal BA 9_L | -0.36617 | 0.0466 |  | 0.59626 | 0.0005 |
| Prefontal BA 10_L | -0.44142 | 0.0146 |  | 0.46148 | 0.0103 |
| Inf. Frontal BA 45_L | -0.43271 | 0.0169 |  | 0.47053 | 0.0087 |
| Postcentral_R | -0.4263 | 0.0188 |  | 0.41765 | 0.0216 |
| Calcarine_R | -0.12076 | 0.525 |  | 0.41003 | 0.0244 |
| Hypo-connectivity ROIs in SAL | | | | | |
| Insula_L | 0.48127 | 0.0071 |  | -0.49501 | 0.0054 |
| Cingulum_Mid_R | 0.40356 | 0.027 |  | -0.38123 | 0.0377 |
| Temporal_Mid_R | 0.41024 | 0.0243 |  | -0.277 | 0.1384 |
| Premotor_R | 0.10875 | 0.5673 |  | -0.34496 | 0.0619 |
| Frontal_Inf_Orb BA 47_R | 0.16462 | 0.3847 |  | -0.37873 | 0.039 |
| Hippocampus_R | 0.47762 | 0.0076 |  | -0.67482 | <.0001 |
| SupraMarginal_R | 0.16946 | 0.3707 |  | -0.46394 | 0.0098 |

| **Table S3. The p values for Multiple Comparisons of Correlation Coefficients of Healthy Controls with Carotid Stenosis Patients before, 1 Month and 1 Year after Carotid Artery Stenting (CAS)** | | | |
| --- | --- | --- | --- |
|  | Before CAS vs Controls | 1 month after CAS vs Controls | 1 year after CAS vs Controls |
| Hyper-connectivity ROIs in SMN | | | |
| Pallidum, R | <0.0001* | 0.0003* | 0.027 |
| Putamen, L | <0.0001* | 0.0086* | 0.0645 |
| Caudate, L | <0.0001* | 0.0016* | 0.0783 |
| Premotor, L | <0.0001* | 0.0394 | 0.0961 |
| Thalamus, L | <0.0001* | 0.0006* | 0.0093* |
| Hypo-connectivity ROIs in SMN | | | |
| Premotor, R | 0.0003* | 0.0075* | 0.2802 |
| Occipital_Sup, R | 0.0002* | 0.1325 | 0.1839 |
| Precuneus, R | 0.0002* | 0.0037* | 0.0288 |
| Hyper-connectivity ROIs in SAL | | | |
| Prefrontal, BA 9, L | <0.0001* | 0.0224 | 0.0383 |
| Prefrontal, BA 10, L | <0.0001* | 0.2069 | 0.0473 |
| Inf. frontal, BA 45, L | <0.0001* | 0.0643 | 0.0551 |
| Postcentral, R | <0.0001* | 0.009* | 0.0803 |
| Calcarine, R | 0.0003* | 0.0094* | 0.0628 |
| Hypo-connectivity ROIs in SAL | | | |
| Insula, L | <0.0001* | 0.2862 | 0.0448 |
| Cingulum_Mid, R | <0.0001* | 0.0019* | <.0001* |
| Temporal_Mid, R | <0.0001* | 0.0058* | 0.0894 |
| Premotor, R | <0.0001* | 0.0012* | 0.0087* |
| Inf. Orb. frontal, BA 47, R | <0.0001* | 0.0632 | 0.0071* |
| Hippocampus, R | <0.0001* | 0.0003* | 0.6123 |
| SupraMarginal, R | <0.0001* | 0.0053* | 0.0003* |
| Alpha is corrected for the multiple t-tests by the Bonferroni method, which is approximately 0.017. | | | |
| * P value below the adjusted alpha.  ROIs indicates regions of interest; SMN, sensorimotor network; SAL, salience network. | | | |
